# Biopsychosocial and clinical characteristics in patients with resected breast and colon cancer at the beginning and end of adjuvant treatment

**DOI:** 10.1186/s12885-019-6358-x

**Published:** 2019-11-26

**Authors:** Teresa García-García, Alberto Carmona-Bayonas, Paula Jimenez-Fonseca, Carlos Jara, Carmen Beato, Beatriz Castelo, Montserrat Mangas, Eva Martínez de Castro, Avinash Ramchandani, David Gomez, Caterina Calderón

**Affiliations:** 1 Department of Medical Oncology, Hospital Santa Lucía, Cartagena, Spain; 2 Department of Medical Oncology, Hospital Universitario Morales Messenger, Murcia, Spain; 3 Department of Medical Oncology, Hospital Universitario Central de Asturias, Oviedo, Spain; 4 Department of Medical Oncology, Hospital Universitario Fundación Alcorcón- Universidad Rey Juan Carlos, Madrid, Spain; 5 Department of Medical Oncology, Hospital Universitario Virgen de la Macarena, Sevilla, Spain; 6 Department of Medical Oncology, Hospital Universitario La Paz, Madrid, Spain; 7 Department of Medical Oncology, Hospital Galdakao-Usansolo, Galdakao-Usansolo, Spain; 8 Department of Medical Oncology, Hospital Universitario Marqués de Valdecilla, Santander, Spain; 9 Department of Medical Oncology, Hospital Universitario Insular de Gran Canaria, Las Palmas, Spain; 10 Department of Clinical Psychology and Psychobiology, Faculty of Psychology, University of Barcelona, Barcelona, Spain

**Keywords:** Healthcare, Patient-centered care, Breast cancer, Adjuvant therapy, Psycho-oncology

## Abstract

**Background:**

The aim of this study was to analyze biopsychosocial factors affecting how patients cope with cancer and adjuvant treatment and to appraise psychological distress, coping, perceived social support, quality of life and SDM before and after adjuvant treatment in breast cancer patients compared to colon cancer patients.

**Methods:**

NEOcoping is a national, multicenter, cross-sectional, prospective study. The sample comprised 266 patients with colon cancer and 231 with breast cancer. The instruments used were the Brief Symptom Inventory (BSI), Mini-Mental Adjustment to Cancer (Mini-MAC), Multidimensional Scale of Perceived Social Support (MSPSS), Shared Decision-Making Questionnaire-Patient (SDM-Q-9) and Physician’s (SDM-Q-Doc), and the European Organization for Research and Treatment of Cancer Quality of Life Questionnaire (EORTC-QLQ).

**Results:**

Breast cancer patients reacted worse to the diagnosis of cancer with more symptoms of anxiety, depression, and somatization, and were less satisfied with their involvement than those with colon cancer (*p* = 0.003). Participants with colon cancer were older and had more physical symptoms and functional limitations at the beginning of adjuvant treatment, while there were scarcely any differences between the two groups at the end of adjuvancy, at which time both groups suffered greater psychological and physical effects and scored lower on coping strategies, except for anxious preoccupation.

**Conclusions:**

Breast cancer patients need more information and involvement of the oncologist in shared decision-making, as well as and more medical and psychological support when beginning adjuvant treatment. Both breast and colon cancer patients may require additional psychological care at the end of adjuvancy.

## Background

Indications for adjuvant treatment for cancer are gradually increasing and adjuvancy has a positive impact on reducing recurrence and mortality, albeit at the expense of greater risk of toxicity and a temporary or permanent negative impact on quality of life. This should be contemplated and included in the decision-making process for this type of treatment.

The first visit with the medical oncologist following resection of non-metastatic cancer is such that it can be difficult to explain the suitability of adjuvant treatment. Patients come in with progressive clinical improvement and are generally aware of their diagnosis, although it has usually been expressed in terms of high curative possibility [1]. It falls to the oncologist to open a probabilistic scenario in which both risks and benefits must be calculated and prognostic uncertainty, side effects, and actual benefit for each individual patient [2] described.

Medical oncologists believe that coping style is highly pertinent to making decisions about adjuvant treatment or active follow-up, dealing with treatment side effects, and the anxiety caused by prognostic uncertainty [2]. Likewise, it is commonly felt that treatment decision and probably tolerance are influenced by physician-patient rapport, by how information is communicated, and by patients’ interpretation of what they are told [3]. It is widely assumed that individuals with certain types of cancer, such as breast cancer, cope differently than those with other types, which affects their quality of life and how they experience their situation [4]. Therefore, it is apropos the validity of these assumptions be determined and whether there are other variables that can improve patient-physician shared decision-making (SDM). Similarly, it is essential that other factors be teased out that can contribute to better patients’ acceptance of their situation and the possible side effects of adjuvant treatment, so as not to lose out on the benefits it offers [5].

Finally, we believe that if they exist, differences in coping style among patients with different types of cancer should be detected, so as to plan the most appropriate support for each, as well as taking into consideration the peculiarities of every type of cancer that can impact such coping. Breast cancer is diagnosed when patients are still young, around the age of 50. This makes the emotional impact even greater at a time when they tend to be the healthy and independent and when their family and professional lives are of great importance [6]. Moreover, adjuvant treatment for breast cancer tends to be more aggressive than for colon cancer; as such, sequelae are potentially greater, last longer, and are more likely to interfere with their job and social interactions. Changes in physical appearance (hair loss, mastectomy, weight gain due to hormone therapy), the decline of their overall physical condition, mainly due to adverse treatment effects, and the uncertainty surrounding prognosis mean that they are more likely to experience psychological distress than patients with other types of tumors such as colon, which is typically diagnosed after the age of 60 [6]. A study by Gibbons et al. [7] and a recent meta-analysis [8] show that coping strategies in breast cancer mediate the relationship between illness perceptions and adjustment to illness. Colon cancer patients have fewer short- and long-term complaints about their quality of life and fewer sequelae than patients with other digestive tumors and breast cancer [9].

The aim of this study was to analyze the biopsychosocial factors that affect how patients cope with cancer and its adjuvant treatment and to appraise psychological distress, coping, perceived social support, quality of life and SDM before and after adjuvant treatment. Based on the hypothesis that breast cancer patients have their own way of coping with the disease and treatment that does not depend on objective factors of severity, prognosis, or toxicity, we focus first on breast cancer patients and then compare them to colon cancer patients.

## Methods

### Study design

NEOcoping is a prospective, multicenter, cross-sectional study of the Continuous Care Group of the Spanish Society of Medical Oncology (SEOM). It was conducted between July 2015 and July 2017 by cancer patients and medical oncologists from 15 Medical Oncology departments in Spain. All participants signed consent forms prior to inclusion in the study. Patients completed self-report scales at baseline, i.e., in the week following the first visit to the Department of Medical Oncology to decide on adjuvant treatment and at the end of adjuvant treatment. The protocol was approved by the Ethics Committee of each hospital and the Spanish Medicines and Health Products Agency (AEMPS). The study was conducted in accordance with the guidelines of Strengthening the Reporting of Observational studies in Epidemiology (STROBE) [10].

### Patients

Patients ≥18 years of age with non-advanced breast and colon cancer treated with surgery with curative intent were eligible. In all cases, the indication for adjuvant chemotherapy was based on international clinical guidelines. Patients with any serious mental illness that prevented them from understanding the study and those treated with preoperative radio- or chemotherapy, only hormonotherapy, or adjuvant radiotherapy without chemotherapy were excluded.

### Measures

The medical oncologist who cared for each patient collected and updated the data through a web-based platform (www.neocoping.es). The demographic and clinical variables recorded are listed in Table 1. The time to diagnosis is the time elapsed from the debut of cancer symptoms to diagnosis. The questionnaires completed are listed in Table 2 and described below.

**Table 1:** Sociodemographic and clinical characteristics of the entire sample and of the patients with colon and breast cancer who went to the Medical Oncology department to decide on adjuvant treatment after curative resection of the cancer

Demographic and clinical characteristics	TOTAL (*n* = 497)	Colon cancer (*n* = 266)	Breast cancer (*n* = 231)	t/χ2	*p*
Gender: n (%)					
Women	337 (67.8)	111 (58)	231 (100)	178.288	0.001
Men	160 (34.5)	155 (42)	0		
Age (years): mean (SD)	58.4 (12.1)	63.1 (11.1)	52.9 (11.0)	9.791	0.001
Marital Status:					
Married/ partnered: n (%)	342 (68.8)	194 (72.9)	148 (72.9)	2.970	0.085
Educational level: n (%)					
Basic	362 (72.8)	206 (84.7)	156 (76.8)	4.546	0.033
Intermediate	135 (27.2)	60 (15.3)	75 (23.2)		
Unemployed: n (%)	281 (56.5)	163 (61.2)	88 (38.0)	10.462	0.001
Tumor stage: n (%)					
I-II	297 (59.8)	89 (33.5)	208 (90.1)	204.075	0.001
III	200 (40.2)	177 (66.5)	23 (9.9)		
Time to diagnosis (days): mean (SD)	176 (99)	192 (107)	156 (83)	2.213	0.027
Type of adjuvant treatment					
Chemotherapy	331 (66.6)	255 (95.8)	76 (32.9)	220.344	0.001
Chemotherapy and radiotherapy	166 (33.4)	11 (4.2)	155 (67.1)		
Risk of relapse (%): mean (SD)	37.6 (37.6)	43.8 (21.1)	30.4 (15.8)	7.859	0.001

*Abbreviations*: *n* number, *SD* standard deviation

**Table 2:** Differences in the impact of cancer, coping strategies, social support, quality of life, and shared decision making by type of cancer, *— colon or breast —* in patients who went to the Medical Oncology department to consider adjuvant treatment

	TOTAL (*n* = 497)	Colon cancer (*n* = 266)	Breast cancer (*n* = 231)	t	*p*
Psychological distress (BSI-18)^a^	63.5 (6.8)	62.4 (6.5)	64.9 (6.9)	−3.903	0.001
Anxiety	61.9 (7.7)	60.8 (7.4)	63.2 (5.7)	−3.218	0.001
Depression	60.4 (5.9)	59.5 (5.7)	61.4 (6.1)	−3.400	0.011
Somatization	60.8 (6.7)	59.9 (6.3)	61.8 (6.9)	−2.978	0.003
Coping with cancer (M-MAC)^b^					
Adaptive	65.7 (17.3)	65.7 (17.3)	66.6 (14.7)	−0.595	0.552
Fighting spirit	78.8 (17.7)	78.5 (17.6)	79.1 (17.7)	−0.385	0.701
Cognitive Avoidance	52.3 (25.9)	51.6 (26.3)	53.1 (25.3)	−0.625	0.532
Fatalism	67.2 (18.9)	66.9 (20.1)	67.5 (17.5)	−0.311	0.756
Maladaptive	32.6 (39.4)	30.2 (19.2)	30.10 (18.8)	0.108	0.914
Helplessness	18.2 (18.2)	19.3 (18.6)	16.7 (17.7)	1.479	0.140
Anxious preoccupation	42.1 (25.5)	41.1 (25.4)	43.3 (25.7)	−0.893	0.372
Social support (MPSS)^c^					
Family	25.7 (3.5)	26.2 (2.9)	25.2 (4.1)	3.089	0.002
Friends	23.6 (4.8)	22.9 (5.1)	24.8 (4.3)	−3.672	0.001
Significant other	25.6 (3.8)	25.8 (3.7)	25.4 (3.9)	1.106	0.269
Quality of life (EORTC QLQ-C30)^b^					
Functional scale	82.6 (11.3)	83.6 (11.1)	81.3 (11.4)	−2.183	0.030
Symptom scale	82.7 (11.7)	84.1 (10.5)	80.9 (12.8)	−2.829	0.005
Health status Scale	56.8 (19.7)	56.6 (19.3)	57.1 (20.3)	−0.155	0.877
General quality of life scale	74.4 (12.4)	74.3 (12.2)	74.1 (12.7)	0.466	0.642
Shared Decision Making (SDM)^b^					
Physician (SDM-Q-Doc)	91.3 (9.1)	91.8 (8.5)	90.7 (90.8)	1.296	0.196
Patient (SDM-Q-9)	82.2 (19.3)	84.5 (17.8)	77.8 (20.7)	2.938	0.003

*Abbreviations*: *BSI* Brief Symptom Inventory, *EORTC-QLQ-C30* European Organization for Research and Treatment of Cancer Quality of Life Questionnaire, *M-MAC* Mini-Mental Adjustment to Cancer, *MSPSS* Multidimensional Scale of Perceived Social Support, *SDM* Shared Decision Making

^a^ T score. ^b^ Scale from 0 to 100. ^c^ Scale from 4 to 28 for each source of support

#### Mini-mental adjustment to Cancer (mini-MAC)

The Mini-MAC is a 29-item scale and assesses cancer-specific coping strategies as adaptive (cognitive avoidance, fighting spirit, and fatalism) or maladaptive (helplessness and anxious preoccupation) [11, 12]. Each item is rated on a 4-point Likert scale ranging from 1 (definitely does not apply to me) to 4 (definitely apply to me). Raw score is transformed into a percentage with 0 indicating the lowest possible level of cancer-specific coping strategies and 100 indicating the highest. Cronbach’s alpha coefficients for each domain ranged from 0.62–0.88 [12, 13].

#### Brief symptom inventory (BSI)

The BSI-18 consists of 18-item divided into three dimensions (somatizations, depression, and anxiety), as well as a total score, the Global Severity Index (GSI), which summarizes the respondent’s overall emotional adjustment or psychological distress over the last 7 days [14]. Raw scores are converted to T-scores based on gender-specific normative data [14], higher scores indicating greater psychological distress. Each item is rated on a 5-point Likert scale. Cronbach’s alpha varied from 0.81 to 0.90 [14].

#### Shared Decision-Making Questionnaire-Patient (SDM-Q-9) and Physician’s (SDM-Q-doc) versions

The SDM-Q-9 and SDM-Q-Doc are short and accurate questionnaires that evaluate the SDM process from the patient’s [15] and physician’s perspective [16]. Each questionnaire contains 9-item, each describing one step of the SDM process [17]. Items are scored from 0 to 5 on a 6-point Likert scale. A total raw score between 0 and 45 is possible. Raw score is transformed into a percentage with 0 indicating the lowest possible level of SDM and 100 indicating the highest. Test-retest reliability was between 0.88 and 0.90 [18, 19].

#### Multidimensional Scale of Perceived Social Support (MSPSS)

The MSPSS evaluates social support and includes 12-item related to three sources of social support: family, friends, and significant other [20]. Responses are provided that range from 1 (very strongly disagree) to 7 (very strongly agree), scores on scale range from 12 to 84 with higher scores indicating greater perceived social support. The MSPSS is extensively used in both clinical and non-clinical samples and has been found to be reliable and valid [21].

#### European Organization for Research and Treatment of Cancer (EORTC) Quality of Life Questionnaire (EORTC-QLQ-C30)

EORTC QLQ-C30 is commonly employed in Europe and its validity has been well established [5, 22]. The 30-item comprise four subscales, ‘Functioning, ‘Symptom, ‘Health Status‘, and ‘General Quality of Life’ and are scored from 1 to 4. Scores for each dimension range from 0 to 100 (0, minimum quality of life; 100, maximum). Higher functional scale, global health status and general quality of life scores and lower symptoms scale scores indicate better QoL.

### Statistical analyses

Demographic data and survey responses were analyzed with descriptive statistics using absolute frequencies for categorical data, mean and standard deviation (SD) for quantitative data and obtaining information from the entire sample and grouped by type of cancer. Bivariate chi-square and t tests were used to assess differences between colon and breast cancer in terms of socio-demographic, clinical and psychological characteristics (impact of cancer, coping strategies, social support, quality of life and SDM). Analysis of the variance of repeated measures was performed on the basis of scores obtained before starting and after finishing adjuvant treatment (impact of cancer, coping strategies and quality of life). These analyses were age-adjusted. Cohen’s effect size (d) and eta squared (η^2^) were used to assess the degree to which differences in continuous variables were associated with group status (breast or colon cancer). Cohen’s d was reported as an indicator of effect size of the differences, with d > 0.2 representing a small, d > 0.5 a medium and d > 0.8 a large effect size [23] and eta-squared ranges between 0 and 1, with η^2^ ~ 01 for a small, η^2^ ~ .06 for a medium and η^2^ > .14 for a large effect size [24]. The data were analyzed using SPSS version 23.0 (IBM Corp., Armonk, NY, USA).

## Results

**Fig. 1:**
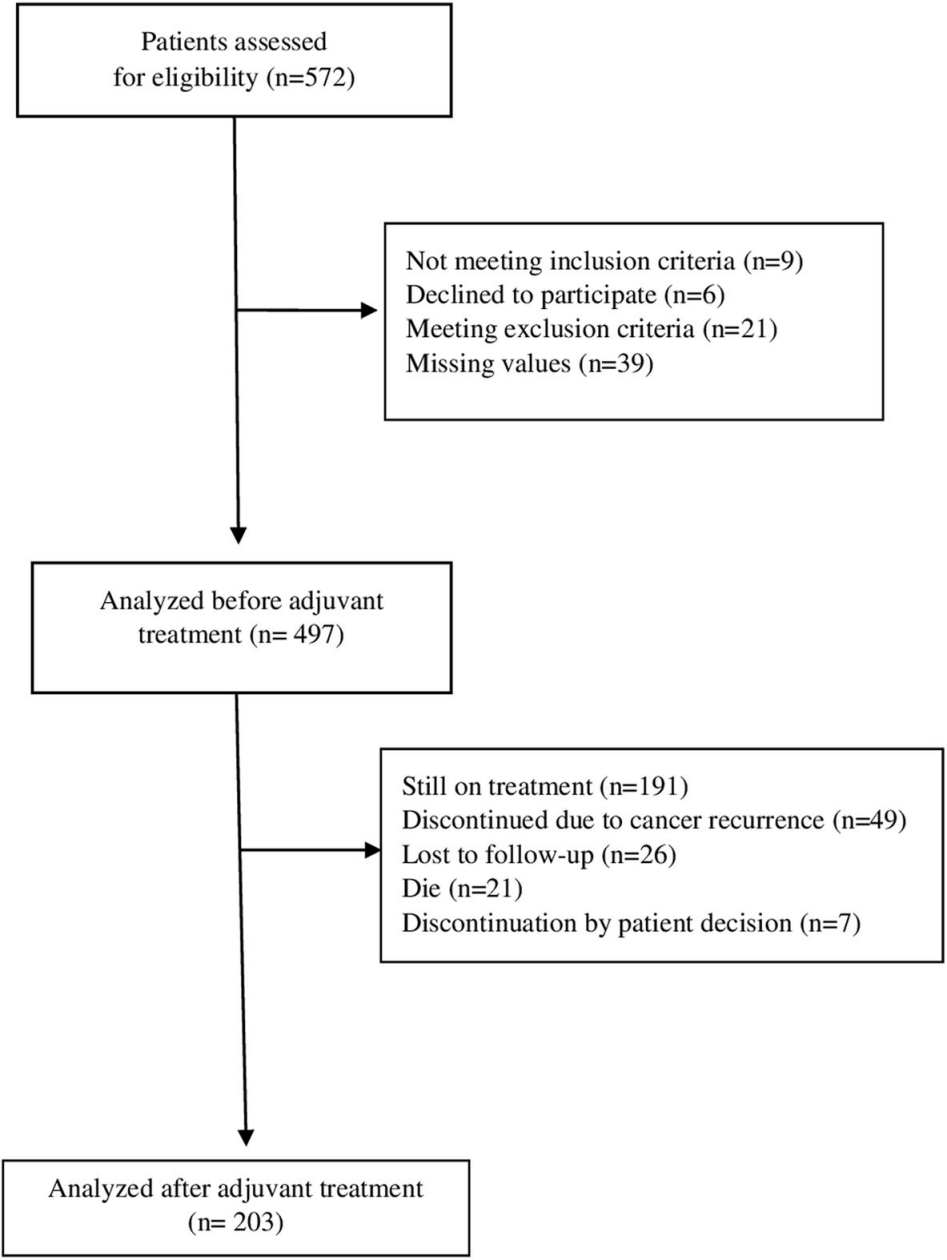
Flow Diagram of the NEOcoping study

We screened 572 patients; 497 were eligible for this analysis and 75 were excluded as shown in the flow diagram, Fig. 1. Socio-demographic and baseline clinical characteristics are presented in Table 1. Two hundred and sixty-six (266) patients had colon cancer [75% stage III and 25% stage II with some factor associated with risk of recurrence] and 231 had breast cancer [stage I-III with criteria indicating adjuvant chemotherapy]. The type of surgery for breast cancer was mastectomy in 42 patients (18%) and lumpectomy in the remaining 82%. The mean age of patients with colon and breast cancer was 63.1 and 52.9 years, respectively. Breast cancer patients had a higher educational level than those with colon cancer. Although the proportion of patients who did not work was high in both groups, it was higher among patients with colorectal cancer mainly because, since they were older, more of them were retired, whereas among the breast cancer subjects, 38% were unemployed. Patients with colon cancer were diagnosed later, had more advanced disease with increased risk of relapse, and treatment tended to consist of surgery and adjuvant chemotherapy, while the most common treatment for breast cancer patients was surgery, chemotherapy, and radiotherapy.

### Psychological adaptation to cancer

Differences in psychological adaptation by cancer type are reported in Table 2. 

#### Cancer diagnosis

The impact of the cancer diagnosis was less negative among colon cancer patients, these patients presented less psychological distress than breast cancer patients (*t*_(441)_ = − 3.903, *p* < 0.001, Cohen’s *d* = 0.372). Breast cancer patients experienced the diagnosis more negatively, with more symptoms of anxiety, depression, and somatization than colorectal cancer patients based on the BSI-18 score. At the beginning of treatment, there were no differences between groups in terms of quality of life, although more physical symptoms (*p* = 0.005, Cohen’s *d =* 0.204) and functional limitations (*p* = 0.030, Cohen’s *d =* 0.042) were reported among colon cancer sufferers.

#### Coping with cancer

Individuals with colon and breast cancer used adaptive strategies. Of the five coping patterns identified, most used fighting spirit and fatalism, while hopelessness was the least common. Though coping patterns were similar, each group looked for social support from different sources. Patients with breast cancer sought more support among friends (*p* < 0.001, Cohen’s *d =* 0.402), whereas participants with colon cancer turned more to relatives for support (*p <* 0*.*001, Cohen’s *d =* 0.281).

#### Shared decision-making

Oncologists were very satisfied with SDM and felt that they informed their patients appropriately without significant differences between oncologists who treat breast or colon cancer. However, participants with breast cancer were less satisfied than those with colon cancer (*p* = 0.003, Cohen’s *d =* 0.347), probably because they wanted to participate more actively in SDM.

### Psychological change after adjuvant treatment

We report data on 114 patients with colon cancer and 89 with breast cancer; i.e., those who had completed adjuvant treatment at the time of this analysis (Fig. 1).

**Fig. 2:**
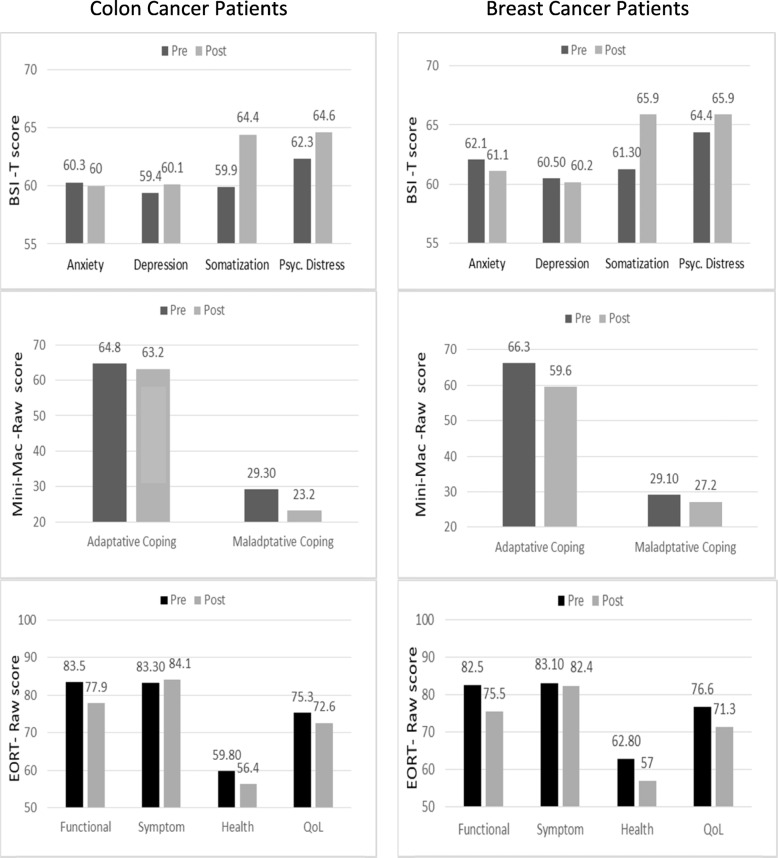
BSI-18, Mini-MAC and EORT-QLQ-C30 scales before and after adjuvant treatment applied to 203 of the patients

To examine the change in psychological parameters resulting from the effect of chemotherapy, the BSI-18, Mini-MAC, and EORTC-QLQ-C30 scales were completed before and after adjuvant treatment and a variance analysis of repeated measures was performed considering pre- and post-adjuvancy scores (see Table 3 and Fig. 2).

**Table 3:** Multivariate analysis of repeated measures before and after adjuvant treatment in patients with colon and breast cancer and curative surgery for the cancer

Scales	Colon cancer (*n* = 114)		Breast cancer (*n* = 89)		ANOVA results, F		
	Pre Mean (SD)	Post Mean (SD)	Pre Mean (SD)	Post Mean (SD)	Time x tumor	Time	Tumor site
Psy. distress (BSI) ^a^	62.3 (6.4)	64.6 (6.7)	64.4 (7.4)	65.9 (6.8)	0.295	11.223^**^	4.691
Anxiety	60.3 (7.1)	60.0 (7.2)	62.1 (8.3)	61.1 (7.6)	1.662	0.414	2.426
Depression	59.4 (5.5)	60.1 (5.7)	60.5 (6.2)	60.2 (5.7)	1.246	0.248	0.845
Somatization	59.9 (6.1)	64.4 (7.2)	61.3 (6.7)	65.9 (7.1)	0.034	80.033^**^	3.112
Coping with cancer (M-MAC) ^b^							
Adaptive	64.8 (18.1)	63.2 (19.5)	66.3 (14.2)	59.6 (17.8)	4.199	11.602^**^	0.205
Fighting spirit	77.2 (18.2)	72.4 (20.9)	77.7 (16.9)	71.8 (21.2)	0.160	13.826^**^	0.002
Cognitive Avoidance	51.4 (26.6)	52.3 (28.1)	53.1 (25.2)	48.7 (25.3)	2.056	0.916	0.084
Fatalism	35.8 (20.6)	63.7 (22.3)	38.7 (17.9)	58.7 (22.1)	6.039	13.707^**^	0.165
Maladaptive	29.3 (18.5)	23.2 (18.2)	29.1 (19.3)	27.2 (20.3)	2.606	9.179^**^	0.612
Helplessness	20.1 (19.1)	13.7 (17.5)	16.1 (17.6)	14.3 (19.5)	2.402	7.509^**^	0.610
Anxious preoccupation	38.3 (22.9)	32.5 (22.5)	42.0 (26.0)	39.6 (24.7)	1.203	6.904^**^	3.124
Quality of life (EORTC-QLQ-C30) ^b^							
Functional scale	83.5 (11.4)	77.9 (13.7)	82.5 (10.4)	75.5 (14.3)	0.572	49.525^**^	1.046
Symptom scale	83.3 (10.3)	84.1 (13.4)	83.1 (13.4)	82.4 (11.7)	0.758	0.008	0.412
Health Status scale	59.8 (18.1)	56.4 (19.4)	62.8 (17.1)	57.0 (20.3)	0.494	7.065^**^	0.710
Quality of life scale	75.3 (11.1)	72.6 (12.1)	76.6 (10.6)	71.3 (12.6)	1.238	16.877^**^	0.021

These analyses were age-adjusted.

*Abbreviations*: *BSI* Brief Symptom Inventory, *EORTC-QLQ-C30* European Organization for Research and Treatment of Cancer Quality of Life Questionnaire, *M-MAC* Mini-Mental Adjustment to Cancer, *Pre* before adjuvant treatment, *Post* after adjuvant treatment, *Psy* psychological, *SD* Standard Deviation

^*^p < 0.01 (two-tailed), ^**^ *p* < 0.001 (two-tailed)

^a^ T score. ^b^ Scale from 0 to 100

Both breast and colon cancer patients reported more somatic symptoms (*p* = 0.001, η^2^ = 0.285), and greater psychological distress after completing treatment (*p* = 0.001, η^2^ = 0.057), with no differences on the basis of the primary tumor.

In terms of post-adjuvancy coping strategies, both groups displayed fewer adaptive (F_(1,196)_ = 11.602, *p = 0.001*, η^2^ = 0.056) and maladaptive strategies (*p* = 0.003, η^2^ = 0.045). Insofar as adaptive strategies are concerned, both groups decreased fighting spirit (*p = 0.001*, η^2^ = 0.066) and fatalism (*p = 0.001*, η^2^ = 0.065), while cognitive avoidance remained stable. As regards maladaptive strategies, both groups exhibited less hopelessness (*p = 0.007*, η^2^ = 0.037) and anxiety (*p = 0.009*, η^2^ = 0.034). No differences were found according to tumor type.

In relation to the quality of life, most patients stated that they suffered a significant decrease in their quality of life after adjuvant treatment on the functioning scale (*p = 0.001*, η^2^ = 0.200), health status scale (*p* = 0.006, η^2^ = 0.039), and on the general quality-of-life scale (*p* = 0.001, η^2^ = 0.083). No differences were found by tumor type.

## Discussion

NEOcoping is a prospective, multicenter study of the Continuous Care Group of the Spanish Society of Medical Oncology (SEOM) that brings together 15 Medical Oncology departments in Spanish hospitals and 497 evaluable patients. To the best of our knowledge, this is among the largest series of variables covering different aspects of the process of coping and SDM on the suitability of adjuvant treatment. The study is ongoing, and results will be completed over time.

This study examines breast and colon cancer because they are common tumors, proven to benefit from adjuvant treatment, and with relatively high cure rates. Beaver et al. compared the same cancers and analyzed preferences in decision-making [25]. They found a more active and demanding attitude in breast cancer patients, which coincides with our findings in this regard. Our study, however, investigates a greater number of clinical, psychological, quality of life, and SDM variables over two very different periods of time: first, following curative surgery and prior to initiating adjuvant treatment and second, at the end of treatment.

Our results suggest that breast cancer patients respond worse to their cancer diagnosis, and are less satisfied with their doctors than colon cancer patients. The latter participants were older and had more physical symptoms and functional limitations at the beginning of adjuvant treatment. Previous studies evaluating patient preferences revealed that breast cancer patients are younger, healthier, and have more active or cooperative roles [5, 26] while individuals with colon cancer would like to be informed and involved in decision-making, but do not necessarily want to make autonomous treatment choices and many prefer a more passive role [27]. In addition, the greater psychological impact of breast cancer, with more symptoms of anxiety, depression, and somatization is not only explained by the fact that it affects younger patients, at a time of maximum professional and family activity, but also by significant changes in appearance (hair loss, mastectomy, weight gain due to hormonotherapy), physical symptoms, and uncertainty about prognosis [6, 7].

Another difference between the two groups is social support. Breast cancer patients look for support from friends and colleagues, while subjects with colon cancer are more family-dependent. This may be a simple generational issue related to the younger age of breast cancer patients.

At the end of adjuvant treatment, there are fewer differences between the groups that are most affected both psychologically (with more somatic symptoms and greater psychological distress) and physically (with lower functional and quality-of-life scores). Likewise, both groups score low on coping strategies except for anxious preoccupation in response to what they may perceive as a kind of a sword of Damocles.

Based on these results, it can be hypothesized that patients are better prepared to fight at the start of treatment and are at their worst psychologically once adjuvant treatment has ended. The subsequent scenario is no longer one of fighting, but of repairing damages and returning to their previous activities and roles, for which they do not yet feel ready. This last observation is especially relevant, given that from that point forward, most patients (at least according to the most usual follow-up protocols) will be left without medical supervision for months after a long series of frequent medical visits during adjuvant treatment. Perhaps, on the basis of these results, we should consider psychological intervention precisely at this point in time. A similar methodology was used by Boinon et al., who interviewed 102 women with breast cancer after surgery and at the end of adjuvant treatment, with special attention paid to the influence of social support on perceived well-being [27]. Patients responded to self-report questionnaires assessing psychological adjustment (depressive symptoms and anxiety related to cancer), social participation concerning their illness, and perceived social support (generic and cancer-specific).

### Study limitations

This study is not without its limitations that must be taken into consideration. First of all, the use of self-report, subjective measures cannot accurately reflect patients’ experiences, expectations, and behaviors, as they are limited by response bias (social desirability, inaccurate memory, etc.), which we have attempted to minimize by reminding patients that answers were completely anonymous and that there were no right or wrong answers. Second, we have compared patients with breast and colon cancer, and 100% in stage I-III; therefore, the results may not generalize to patients with other tumors or in stage IV disease. Another limitation, despite sex was taken into consideration in subgroup analyses, is that breast cancer patients were mostly women, while colon cancer patients were men and women. Therefore, characteristics due to sex may have influenced the results. Finally, there are numerous factors that can influence treatment decision and that have not been considered, such as the presence of comorbidities, type of treatment, and side effects.

Given the size of our sample and the prospective nature of the evaluation, we consider the outcomes to be reliable, robust, and relevant, which allows us to suggest measures to improve coping and psychological well-being at the beginning and end of adjuvant chemotherapy. We believe this study to be a benchmark in this field. The limitation of exploring only two-time periods in a continuous process can be overcome with subsequent surveys, such as in the study by Ganz et al. [28] who collected data from 558 women with breast cancer after surgery and at 2.6 and 12 months, revealing significant physical and psychosocial recovery in the first year after treatment had been completed.

## Conclusions

In conclusion, NEOcoping helps us to comprehend how patients with non-metastatic colon and breast cancer cope with cancer before and after adjuvant treatment. Moreover, we have identified aspects that impact quality of life and psychological well-being. The results point toward breast cancer patients needing more information and involvement of the oncologist in SDM and more medical and psychological support initially. Patients with colon and breast cancer suffered greater psychological and physical effects and scored lower on coping strategies, except for anxious preoccupation, after completing treatment. This indicates that we should modify the timing of psychological care for both groups, increasing support at times when, at present, patients are typically left alone; i.e., after the end of adjuvant treatment.

## Data Availability

Research Data are not shared.

## Abbreviations

SDM: Shared Decision Making
STROBE: Strengthening the Reporting of Observational Studies in Epidemiology
